# Armed Conflict and Unwanted Births in Colombia

**DOI:** 10.1177/00221465251353533

**Published:** 2025-07-24

**Authors:** Signe Svallfors

**Affiliations:** 1 Stockholm University, Stockholm, Sweden; 2 Stanford University, Stanford, CA, USA

**Keywords:** armed conflict, Colombia, reproductive justice, sexual and reproductive health and rights, unwanted birth

## Abstract

Armed conflict has been linked to fertility changes globally, but little is known about how reproductive autonomy is affected. Unwanted fertility is likely to occur during conflicts due to escalations of sexual violence and restricted access to contraception and abortion, especially among marginalized groups. Drawing on an intersectional lens of reproductive justice, this study investigates the relationship between women’s exposure to local conflict violence and experiences of unwanted births in Colombia. The study is based on a nationally representative sample of 16,476 children born between 1999 and 2015, from the Demographic and Health Surveys, linked to spatiotemporal conflict data from the Uppsala Conflict Data Program. Results from fixed effects regressions show that exposure to conflict is indeed associated with a higher probability of experiencing unwanted births, especially among socioeconomically disadvantaged people. The study also evaluates how patterns vary by the frequency, intensity, duration, geographical scope, and type of conflict.

The ability to define and realize one’s own reproductive decisions is a cornerstone of rights-based perspectives on health (Senderowicz 2020). It has been well established that sexual and reproductive health (SRH) and rights suffer from armed conflict (Hedström and Herder 2023). War has been linked to various changes in fertility behavior globally, including both postponements and accelerations of the timing of births and both increases and declines in parity progressions (e.g., Castro Torres and Urdinola 2018; Torrisi 2020; Verwimp, Osti, and Østby 2020). Despite growing interest from academics and policymakers in the impact of armed conflict on women’s SRH, prior research has not fully disentangled intentional from haphazard changes in fertility behavior. In other words, we know little about whether fertility changes during conflict were wanted.

The few existing studies that have empirically investigated how war shapes the proximate determinants of fertility, such as fertility intentions and regulation, report mixed findings. Some observe lower contraceptive use among conflict-affected women, and others have reported an acceleration in women’s first contraceptive use, suggesting a preference for postponing first births (Svallfors and Billingsley 2019; Torrisi 2024; Williams et al. 2012). Research on fertility desires, preferences, and ideals shows null or positive relationships, suggesting that conflict is sometimes—but not always—linked with replacement effects through which women wish to replace lost kin and rebuild their social communities by having more children (Rutayisire, Broekhuis, and Hooimeijer 2013; Svallfors 2022b).

In Colombia, protracted conflict since the 1960s poses a threat to reproductive well-being and autonomy, as acknowledged in recent peace and reconciliation efforts. Prior research shows that although fertility levels have increased and women have less access to contraception and abortion in more conflict-affected areas, women’s fertility preferences have not changed (Castro Torres and Urdinola 2018; Svallfors 2022a, 2022b; Svallfors and Billingsley 2019). This suggests that conflict may have created a surge in unwanted fertility in this context.

In this study, I analyze the association between Colombian women’s exposure to armed conflict and their likelihood of experiencing an unwanted birth. The study is based on a combination of nationally representative surveys and information about the timing and location of conflict events.

Most prior research on war and population health focuses on main effects, that is, controlling for instead of analyzing how various axes of difference—such as gender, class, and age—shape health outcomes of violence. However, as theorized by intersectionality and reproductive justice scholars, experiences of violence and restrictions to bodily autonomy are disproportionally borne by marginalized people (Collins 2015; Ross and Solinger 2017). Hence, I consider both population-level patterns and intersectional dimensions across sociodemographic groups. Intersectionality is a lens that enables studying contextual determinants of health across heterogeneous population subgroups by examining how the position of individuals within specific power structures affects health (Bauer 2014).

To pinpoint the dimensions of conflict that most impact unwanted fertility, I explore the frequency, intensity, duration, geographical scope, and type of conflict exposure. This examines whether conflict is more salient when considering battle events or casualties, nearby or more distant, and in the immediate, intermediate, or long term. Differentiating between conflict types addresses whether violence matters more when the victims are civilians, when battle events involve state or nonstate armed groups, or irrespective of who committed and suffered from violence. Although common within the broader conflict literature (Davies, Pettersson, and Öberg 2023), the distinction between conflict types has seldom been used to study health outcomes of war.

Colombia is an especially suitable case for studying the relationship between conflict and unwanted fertility, owing to its high-quality, high response rate and highly detailed household surveys. Despite its uniquely long-standing conflict, data collection has continued virtually uninterrupted, which is rare in highly violent settings. The case of Colombia also allows for studying the impact of conflict on reproductive autonomy and well-being in a low-fertility setting where contraceptive knowledge and use are highly prevalent, although reproductive health metrics are highly stratified (Sedgh and Hussain 2014). Colombia is currently undergoing a gender-inclusive peace and reconciliation process aiming for total peace. The findings from this study can be used to inform transitional justice and conflict reparations, comprehensive family planning programs, and other SRH interventions serving war-affected people in Colombia and beyond.

## Unwanted Fertility as an Indicator of Reproductive Autonomy

Unwanted and unintended pregnancy and fertility are central concepts in population health and demographic research to explain the disconnect between fertility preferences, intentions, and behavior. Planned childbearing can be achieved through sexual activity (e.g., avoiding sex during ovulation), contraception, and abortion. The widespread and rapid adoption of contraception in the second half of the twentieth century has led to a substantial fertility decline globally, but reports of unwanted births suggest that people are still unable to fully implement their preferences (Bongaarts 1997). Although global rates of unintended pregnancy and fertility have declined over the past decades, the magnitudes of declines vary considerably between regions (Bearak et al. 2020; Casterline and El-Zeini 2022). Globally in 2015 to 2019, an estimated 121 million unintended pregnancies occurred per year, out of which 39% ended in unplanned births (Bearak et al. 2020).

The right to determine whether, when, and with whom to have children is fundamental to health and well-being. Unwanted pregnancies and births have been widely—although not uniformly—linked to adverse health and socioeconomic outcomes (Gipson, Koenig, and Hindin 2008; Musick et al. 2009). Unplanned pregnancies can also be wanted, and fertility preferences may be ambivalent and subject to change over time (Sennott and Yeatman 2018; Trinitapoli and Yeatman 2011), for example, in response to external influences, such as conflict (Rutayisire et al. 2013; Svallfors 2022a; Svallfors et al. 2024).

## Intersectional Dimensions of Reproductive Health and Armed Conflict

From a reproductive justice perspective, the links between exposure to local armed conflict and unwanted fertility speak to the importance of sociopolitical context for shaping bodily autonomy and reproductive health. Reproductive justice theory holds that people in marginalized positions more often encounter barriers to health care and autonomy, related to gendered, classed, and racialized power dynamics of the particular setting (Ross and Solinger 2017). Armed conflict often takes a significant toll on women’s ability to exercise free choice when it comes to body, sexuality, and reproduction (Hedström and Herder 2023). There are three primary reasons why we may expect conflict to increase the risk of unwanted birth: (1) reduced access to health care services, (2) increased exposure to sexual violence, and (3) links between intrauterine exposure to violence and adverse infant health outcomes. Specifically, (1) conflict may limit contraceptive use due to wartime limitations in health care provision, worse economic conditions of individuals and households, and reduced bodily autonomy (Haar et al. 2021; Svallfors 2024c; Svallfors and Billingsley 2019; Torrisi 2024); (2) conflict is also a known driver of sexual violence, perpetrated by both intimate partners and armed groups, placing women at risk of unwanted births, especially in places with limited access to safe and legal abortion (Svallfors 2023, 2024c; Torrisi 2023); and (3) intrauterine exposure to conflict has been established as a risk factor for numerous detrimental pregnancy outcomes, such as low birth weight, pregnancy loss, stillbirth, and prematurity (Svallfors et al. 2025; Torche and Nobles 2024). These findings may be partially related to unwanted births, which have been linked to adverse infant health outcomes per se (Gipson et al. 2008).

Reproductive health can be affected by conflict both directly, due to armed actors perpetrating sexual violence and limiting free mobility for political purposes, and more indirectly, due to faltering health infrastructure and economic circumstances that sustain reproductive health and autonomy (Svallfors 2024c). Such consequences are likely intersectional, reflecting patterns of structural violence and social inequality (Ross and Solinger 2017).

Intersectionality is a key concept for this study, defined here as the recognition that dimensions of oppression are systematically embedded in mutually reinforcing social structures (Collins 2015). Intersecting identities related to attributes such as income, location, education, ethnicity, and gender influence how individuals experience and cope with contextual stressors (Ross and Solinger 2017). The impact of war on reproductive health is likely to vary depending on the sociodemographic characteristics of the people who are exposed to violence. In Colombia, ethnic minority, poor, and low-educated women in rural areas have been particularly deprived of health care while also more exposed to sexual violence, suggesting multiplicative burdens on reproductive health (Svallfors 2024c). For example, socioeconomically disadvantaged people in remote and marginalized areas of the country have faced particular challenges in accessing contraception due to both preexisting and conflict-exacerbated vulnerabilities (Svallfors 2024c). The intersectional lens of reproductive justice is useful for understanding how conflict intersects with inequity dimensions, structural forces, and institutions in shaping SRH outcomes. This is particularly important for places such as Colombia, where health, access to health care, and gender equality are ridden with social inequity.

## Exposure to Armed Conflict

Despite growing academic and policy interest in the health consequences of armed conflict, there are no standard definitions of conflict exposure. Prior studies use a variety of indicators, sometimes without clear descriptions of data decisions. Hence, existing literature provides little theoretical or empirical guidance for how health outcomes vary by different forms of exposure to conflict.

Political science research often distinguishes between types of conflict as (1) state-based violence involving the state and one or more rebel groups; (2) nonstate violence between organized actors, neither of which is the state government; and (3) one-sided violence by armed groups against civilians (Davies et al. 2023). This typology has rarely been applied in health research despite plausible differences. For instance, violence against civilians likely has a distinct effect due to fear of being personally affected, knowing victims, and witnessing violence (Carpenter 2006). Meanwhile, state-based conflict might involve more organized and better equipped actors inflicting great damage on health care infrastructure (Østby et al. 2018).

Furthermore, the effects of violent exposure likely vary by the frequency (the number of events), the intensity (the number of casualties in events), and the duration of conflict. Plausibly, a single massacre may be highly impactful on the immediate and long-term psychosocial well-being of affected communities, whereas frequent killings over long periods could be more detrimental to the health care system’s ability to retain staff, receive shipments, and keep clinics open (Haar et al. 2021). Temporal differences may also arise; exposure to conflict could have both immediate effects, such as trauma and stress, and long-term effects on health care provision and essential food, water, or waste infrastructure.

Few studies differentiate between how distinct types of violence affect health outcomes, with two notable exceptions. Williams et al. (2012) found that major district-level gun battles and political events in the previous month accelerated transitions to marriage and first contraception in Nepal, whereas ceasefires correlated with a decrease in marriage but not with contraceptive use. Østby et al. (2018) found that local state-based and one-sided—but not nonstate—violence in Sub-Saharan Africa within a buffer zone of 50 km from survey cluster locations was associated with a lower probability of giving birth in a health care facility. However, the authors cautioned against overinterpreting these differences due to low variation in nonstate violence (Østby et al. 2018). This is not the case in Colombia, where much violence results from battles between guerrillas and paramilitary groups (see the section “Conflict and Health in Colombia”). Hence, the Colombian case is suitable for investigating differences in health effects of exposure across conflict types.

In this study, conflict exposure is conceptualized as living in an area affected by organized conflict violence. A specific contribution of the study is the analysis of how distinct exposures to conflict—varying by the types, frequency, intensity, and duration of exposure—are associated with unwanted births. These distinctions are important to understand when and how conflict is most salient for population health and reproductive autonomy, which, in turn, is critical for designing relevant policies and interventions.

## Conflict and Health in Colombia

After decades of increasing political instability, Colombia’s internal armed conflict ignited in the 1960s when various left-wing guerrilla movements, such as FARC-EP (the Revolutionary Armed Forces of Colombia-People’s Army) and ELN (the National Liberation Army), formed in response to political elitism, clientelism, exclusion of other political views, and socioeconomic injustice. Wealthy landowners then formed paramilitaries, most of which were included in AUC (The United Self-Defense Forces of Colombia), which disbanded in 2006 (Steele 2017). Over the decades, conflict dynamics have grown ever more complex. Narcotrafficking emerged in the 1980s as an income for illicit armed groups, fueling further conflict over key territories and blurring the line between political, economic, and criminal elements of warfare. Violence has been most intense in rural areas and regions, where armed groups have fought over territorial control (Jansson 2008; Steele 2017). Armed groups have used a broad repertoire of violence, varying by form, frequency, and technique, although all have targeted civilians (Gutiérrez-Sanín and Wood 2017). Public displays of excessive atrocities and cruelty—for example, in the form of corpse mutilation and mass killings—have been used to create fear in local communities. However, there has been little violence committed by civilians against other civilians (Ritholtz 2022).

The impact of violence on the lives and health of civilians cannot be understated. Between 1975 and 2004, 554,008 homicides were committed in the country, representing a mean of 1 homicide every 30 minutes and 10% to 15% of the mortality rate. In 1997 to 2002, 930 massacres were recorded—a mean of 1 per day—totaling 2,630 casualties. Systematic use of kidnappings and antipersonnel mines has further added to the public health burden (Franco et al. 2006). Sexual violence has been used as a weapon of war to punish political allegiances and instill fear in the population, an opportunity crime due to impunity and amplified harmful gender norms (Svallfors 2024a, 2024c).

The health care system has been badly affected because of redirected resources to military goals, attacks against health care, and limited state presence in more remote areas (Crawford et al. 2023; Franco et al. 2006; Svallfors 2024c). Consequently, access to reproductive health care—including contraception—has suffered (Ramos Jaraba et al. 2020; Svallfors and Billingsley 2019). Abortion was legalized in Colombia in 2022, following partial decriminalization under certain circumstances in 2006, with limited service availability. Hence, for most of the conflict, safe abortion services were unavailable despite pervasive sexual violence (Daigle, Duffy, and Castañeda 2022; Svallfors 2024a).

The conflict has been linked to various deleterious reproductive outcomes, such as gender-based violence, miscarriage, lower infant birthweight, infant and child mortality, and maternal mortality (Camacho 2008; Castro Torres and Urdinola 2018; Ramos Jaraba et al. 2020; Svallfors 2022a, 2022b, 2023, 2024c; Svallfors and Billingsley 2019; Svallfors et al. 2025).

As posited by reproductive justice theory, war likely affects people differently depending on their social attributes and lived experiences. Despite stable macro finances, Colombia has one of the most unequal income distributions in the world. In 2015, one-third of the country’s population lived in poverty, and one-tenth lived in extreme poverty (DANE 2016). Poor women with lower levels of education in rural areas face a double burden of being disproportionally affected by war and having fewer resources to facilitate reproductive health (Svallfors 2024c). In rural areas only, conflict has been linked to higher fertility (Castro Torres and Urdinola 2018). Moreover, around one-fifth of teenagers become pregnant before the age of 18 and one-sixth before the age of 16 (Martes-Camargo 2015), partly due to conflict (Svallfors 2024b). In Colombia, where sexual violence is pervasive and access to contraception and legal abortion is limited, we can expect a surge in unwanted and mistimed births in response to armed conflict, especially among marginalized groups.

## Data and Methods

This study drew on a combination of individual-level surveys and data on conflict events, leveraging variations in violence across space and time to generate estimates of how women’s exposure to armed conflict correlates with their experiences of unwanted births.

The nationally representative, cross-sectional Demographic and Health Surveys (DHS) have been conducted in Colombia every fifth year since the 1980s, administered by Colombia’s leading family planning organization, Profamilia. The three latest rounds (2004–2005, 2009–2010, and 2015–2016) contained unique retrospective information about each birth to respondents five years before the interview, including whether births were wanted and for how long pregnancies lasted. The DHS sampled girls and women ages 15 to 49 and, in the two latest surveys, girls ages 13 to 14. Response rates were above 90% in each survey.

From the DHS, I created a data set in which each observation was a birth. From an original data set of 133,583 respondents, I removed those who had never given birth (n = 46,452), had no births in the past five years (n = 50,976), or had invalid information on age (n = 89). After expanding the data set to women-births, observations with unclear birth months (n = 15) were removed. To account for migration when assigning exposure, I removed births in past residences (n = 27,572) because information about their location was unavailable. Based on these selection criteria, the sample contained 16,476 births during 1999 to 2015 to 13,873 women.

Data on armed conflict were drawn from the Uppsala Conflict Data Program Georeferenced Event Dataset (UCDP-GED), capturing the timing and location of each event of conflict involving organized armed actors in which at least one person was killed. It only includes events reported in news media, nongovernmental reports, books, or other secondary sources (Davies et al. 2023; Sundberg and Melander 2013). This data set is widely considered the gold standard for measuring subnational conflict violence but provides a conservative measure because it excludes nonlethal and unreported violence (Eck 2012). I removed events without information about the location and month of occurrence.

### Key Variables

The key dependent variable measured *unwanted birth*, defined as whether each birth was wanted. An alternative specification captured whether births were wanted at the time, later, or not at all. This distinction enables an investigation of both unwanted and mistimed births (D’Angelo et al. 2004). For each child born in the past five years, respondents were asked: “At the time you became pregnant, did you want to become pregnant then, did you want to wait until later, or did you not want to have any (more) children at all?” Unwanted births are likely underestimated because data were only available for live births from respondents who survived pregnancy and may be subject to ex post rationalization because of hesitancy to report existing children as unwanted (Mumford et al. 2016).

Colombia has 32 departments and the capital district of Bogotá, both of which I refer to as “departments” for concision, and 1,122 municipalities, which group to form departments. I matched respondents to conflict events by their municipality and department of residence and each month before conception, accounting for gestational length. Gestation was self-reported in monthly format; 83% of pregnancies spanned 9 months, 14% 8 months, and 2% or less 5, 6, 7, or 10 months, respectively.

I then created multiple linear indicators to capture the frequency, intensity, duration, geographical scope, and type of conflict exposure. Frequency measured the number of events, a intensity captured the number of casualties. Duration was measured by summarizing violence in the three months, one year, and five years before conception. Indicators captured violence in both larger departments and smaller municipalities. Conflict type differentiated between state-based, nonstate, and one-sided violence and grouped all violent events.

Akaike’s information criterion (AIC) and *p* values were used to evaluate model fit and the importance of the multiple conflict indicators, net of birth order and conception year. According to AIC, municipality-level indicators consistently outperformed the department level. Only for department-level measures, all *p* values were below the 5% threshold, suggesting a more consistent pattern at more aggregated geographic levels. Nevertheless, municipality-level measures were preferred for model fit and accounting for localized violence. AIC tests and *p* values were agnostic as to whether frequency or intensity fit the data better, except for one-sided violence, where indicators of events contributed more to model fit than casualties. Comparing durations, one- and five-year measures contributed more than one- or three-month lags, indicating that exposure matters more when measured over intermediate or longer durations than in the immediate term. Consequently, the key independent variables were identified as municipality-level conflict frequency and intensity one year and five years before conception. Results from these model fit exercises are in Appendix Tables A1 to A4 in the online version of the article.

### Analytic Approach

The main analysis used a linear probability model (LPM) estimating the probability that births were unwanted. All models included municipality-fixed effects combined with municipality-clustered standard errors to net out any local variation that could codetermine both exposure and outcome (in models to establish model fit of department-level measures of conflict, I used department fixed effects and clustered standard errors). Control variables included *year* of conception, *birth order*, and the respondent’s *age at conception*. Average marginal effects based on the LPMs estimated predicted probabilities of the magnitude of the significant relationships between conflict and unwanted birth, accounting for covariates at their observed values.

To explore heterogeneous effects in the relationship between exposure to conflict and the probability of having an unwanted birth, the main models were partitioned by four separate variables.

First, *teen birth* was defined as the respondent being less than 20 years old at conception (in these models, the age covariate was removed).

Second, *type of place of residence* defined urban as “living in a nucleus of 1,500 or more inhabitants” (Montgomery et al. 2003:491). Three categories were used: rural, urban with low population density (<50 inhabitants per km^2^), and urban with high population density (50+ inhabitants per km^2^). Alternative analyses used the simple rural/urban distinction and indicators separating between urban areas with less than or at least 100 inhabitants per km^2^. The population density data was fixed for 2010 due to data availability.

Third, *educational level* was time-varying and approximated whether respondents had finished primary, secondary, or tertiary school. It was constructed from the highest level of education achieved at the interview, the respondent’s age, the typical age at graduation in Colombia, and whether the respondent was in education at the interview. The latter condition serves to assess whether education was likely finished at that point. This indicator assumes a quite rigid educational system with no study breaks, repeating of school years, or early/postponed entries into the school system.

Fourth, *parity of the child* differentiated between the first, second, or third and higher order (in these models, the birth order covariate was removed).

Finally, to create an intersectional understanding (Scott and Siltanen 2017), I calculated average marginal effects for people at the intersection of the social characteristics most likely to exhibit unwanted births when exposed to conflict.

The decision to stratify the sample rather than use interaction terms to assess heterogeneity is motivated by recent research showing that interaction terms in fixed effects models do not purely capture within-unit variation as intended (Giesselmann and Schmidt-Catran 2020). Instead, estimates incorporate both within-area and between-area variation, potentially reintroducing the area-specific heterogeneity that fixed effects should control for.

## Results

Table 1 displays descriptive statistics of the sample. Around half (48%) of respondents reported their children being wanted at the time of birth, 25% were wanted later, and 27% were not wanted at all. Notwithstanding ex post rationalization, this suggests that unwanted childbearing is a common experience among Colombian women.

**Table 1.** Descriptive Statistics of the Sample Population (*N* = 16,476).

	Frequency/*M*	Proportion/*SD*
Unwanted birth		
Yes	7,917	48%
No	8,559	52%
Unwanted birth (alternative specification)		
Wanted at the time	7,917	48%
Wanted later	4,038	25%
Not wanted at all	4,521	27%
Birth order	1.17	.42
Conception year	2006	4.25
Respondent’s age at conception	24.40	6.65
Teen birth		
No	12,864	78%
Yes	3,612	22%
Type of place of residence		
Rural	4,723	29%
Urban, low density (<50 inhabitants/km^2^)	5,724	35%
Urban, high density (≥50 inhabitants/km^2^)	5,769	35%
Urban, population density missing	260	2%
Education		
Primary	7,541	46%
Secondary	6,727	41%
Tertiary	2,208	13%
Parity of the child		
First	5,311	32%
Second	4,744	29%
Third or higher order	6,421	39%
Exposure to conflict (all types of violence)		
Conflict frequency in the past month	.10	.43
Conflict frequency in the past 3 months	.28	.94
Conflict frequency in the past 1 year	1.17	3.07
Conflict frequency in the past 5 years	6.50	12.00
Conflict intensity in the past 1 month	.29	1.93
Conflict intensity in the past 3 months	.88	3.65
Conflict intensity in the past 1 year	3.68	11.05
Conflict intensity in the past 5 years	20.79	39.90

*Note:* Based on author’s calculations of data from Demographic and Health Surveys and Uppsala Conflict Data Program Georeferenced Event Dataset.

Among births, 22% were to adolescent mothers, 29% in rural areas, 46% to respondents with primary or lower levels of education, and 41% to those with secondary education. The birth order ranged from 1 to 5, with a mean value of 1.17. The mean year of conception was 2006. The age at conception was 24 years on average, ranging from 11 to 47.

Respondents were exposed to an average of 1.17 events (*SD* = 3.07, range = 0–58) and 3.68 casualties (*SD* = 11.05, range = 0–200) in the year before conception and 6.50 events (*SD* = 12.00, range = 0–114) and 20.79 casualties (*SD* = 39.90, range = 0–427) in the five years before conception. Respondents were generally more exposed to state-based violence, followed by one-sided violence, and least to nonstate violence (Appendix Table A5 in the online version of the article).

Table 2 displays four separate LPMs of unwanted births in relation to violent conflict. Each model measured, respectively, the frequency and intensity of conflict in the respondent’s municipality of residence in the year or five years preceding conception. These models adjusted for birth order, the respondent’s age at conception, municipality fixed effects, and municipality robust standard errors.

**Table 2.** Municipality Fixed Effects Linear Probability Models of Unwanted Birth in Relation to Armed Conflict in Colombia (*N* = 16,476).

	Model 1	Model 2	Model 3	Model 4
	*B* (*SE*)	*B* (*SE*)	*B* (*SE*)	*B* (*SE*)
Conflict frequency in the past year	.0036*			
	(.0014)			
Conflict frequency in the past 5 years		.0008		
		(.0005)		
Conflict intensity in the past year			.0008	
			(.0005)	
Conflict intensity in the past 5 years				.0003*
				(.0001)
Constant	.8990	1.6210	1.4031	1.2455
	(2.4150)	(2.4964)	(2.4143)	(2.5428)
*R* ^2^	.01	.01	.01	.01
Rho	.16	.16	.16	.16
AIC	23,094.42	23,096.88	23,096.39	23,096.18

*Note:* Based on author’s calculations of data from Demographic and Health Surveys and Uppsala Conflict Data Program Georeferenced Event Dataset. All models adjust for birth order, the year of conception, and the respondent’s age at conception. Conflict frequency was measured as the number of battle events; conflict intensity was captured as the number of casualties in those events. Exposure to conflict is measured prior to conception. Municipality robust standard errors are in parentheses. AIC = Akaike information criterion.

* *p* < .05.

The findings show that the higher the conflict frequency in the year preceding conception, the higher the probability of unwanted birth (Model 1). A higher intensity of conflict, on the other hand, was significantly linked to unwanted birth when measured five years before conception (Model 4). The probability of unwanted birth was .36 percentage points higher for each additional conflict event during the past year and .03 percentage points higher for each additional casualty during the past five years. The estimates of conflict frequency in the past five years and conflict intensity in the past year were also positive but insignificant (Models 2 and 3). This suggests that conflict exposure in both the intermediate and long-term is associated with unwanted births, although the relationship varies depending on whether events or casualties are counted.

**Figure 1.**
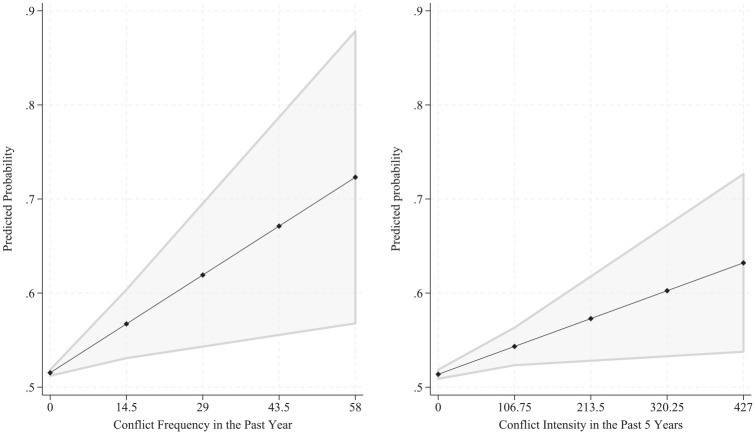
Predicted Probabilities of Unwanted Birth across Levels of Exposure to Armed Conflict Violence in Colombia (N = 16,476). *Note:* Based on author’s calculations of data from Demographic and Health Surveys and Uppsala Conflict Data Program Georeferenced Event Dataset.

Next, I used these models to calculate predicted probabilities of unwanted birth across levels of conflict exposure (Figure 1). Among unexposed women, the predicted probability of unwanted birth was close to the sample mean (around 51 percentage points, confidence interval [CI]) = 51–52). However, among respondents exposed to the highest frequency of conflict in the past year, the predicted probability of unwanted birth was 73 percentage points (at 58 events, CI = 56–88), a 43% higher predicted probability than the unexposed. Similarly, those exposed to the highest intensity of conflict in the past five years had a 64 percentage point probability of unwanted birth (at 427 casualties, CI = 55–72), corresponding to a 25% higher predicted probability than the unexposed.

### Conflict Types and Heterogeneous Effects

Next, I disaggregated the indicators into state-based, nonstate, and one-sided violence. Exposure to all three types of conflict was significantly linked to a higher probability of unwanted birth (Table 3, Models 1, 3, 6, 8, and 10), albeit varying by the duration and definition of exposure (frequency or intensity). It suggests that conflict type is not an important determinant of unwanted births.

**Table 3.** Municipality Fixed Effects Linear Probability Models of Unwanted Birth in Relation to Armed Conflict in Colombia, by Type of Conflict (*N* = 16,476).

	Model 1	Model 2	Model 3	Model 4	Model 5	Model 6	Model 7	Model 8	Model 9	Model 10	Model 11	Model 12
	*B* (*SE*)	*B* (*SE*)	*B* (*SE*)	*B* (*SE*)	*B* (*SE*)	*B* (*SE*)	*B* (*SE*)	*B* (*SE*)	*B* (*SE*)	*B* (*SE*)	*B* (*SE*)	*B* (*SE*)
State-based violence												
Conflict frequency in the past year	.0078***											
	(.0023)											
Conflict frequency in the past 5 years		.0007										
		(.0011)										
Conflict intensity in the past year			.0012*									
			(.0005)									
Conflict intensity in the past 5 years				.0003								
				(.0002)								
Nonstate violence												
Conflict frequency in the past year					.0037							
					(.0146)							
Conflict frequency in the past 5 years						.0093*						
						(.0040)						
Conflict intensity in the past year							–.0008					
							(.0016)					
Conflict intensity in the past 5 years								.0024*				
								(.0011)				
One-sided violence												
Conflict frequency in the past year									.0029			
									(.0026)			
Conflict frequency in the past 5 years										.0017*		
										(.0009)		
Conflict intensity in the past year											.0009	
											(.0010)	
Conflict intensity in the past 5 years												.0004
												(.0003)
Constant	.9678	2.3711	1.5984	1.7091	2.7115	2.2039	2.8852	1.8529	2.0004	1.6134	2.2151	2.1096
	(2.4088)	(2.4761)	(2.4026)	(2.5598)	(2.4936)	(2.4957)	(2.5358)	(2.5089)	(2.4673)	(2.5665)	(2.4222)	(2.6019)

*Note:* Based on author’s calculations of data from Demographic and Health Surveys and Uppsala Conflict Data Program Georeferenced Event Dataset. All models adjust for birth order, the year of conception, and the respondent’s age at conception. Conflict frequency was measured as the number of battle events; conflict intensity was captured as the number of casualties in those events. Exposure to conflict is measured prior to conception. Municipality robust standard errors are in parentheses.

* *p* < .05, ***p < .001

I examined heterogeneous effects according to age (Table 4), residence (Table 5), education (Table 6), and parity (Table 7), grouping all types of conflict.

**Table 4.** Municipality Fixed Effects Linear Probability Models of Unwanted Birth in Relation to Armed Conflict in Colombia, by the Respondent’s Age at Conception (*N* = 16,476).

	Adult (≥20 Years Old)				Teenager (<20 Years Old)			
	Model 1	Model 2	Model 3	Model 4	Model 5	Model 6	Model 7	Model 8
	*B* (*SE*)	*B* (*SE*)	*B* (*SE*)	*B* (*SE*)	*B* (*SE*)	*B* (*SE*)	*B* (*SE*)	*B* (*SE*)
Conflict frequency in the past year	.0033*				.0042			
	(.0015)				(.0030)			
Conflict frequency in the past 5 years		.0008				.0013		
		(.0005)				(.0012)		
Conflict intensity in the past year			.0007				.0010	
			(.0005)				(.0008)	
Conflict intensity in the past 5 years				.0003*				.0003
				(.0001)				(.0004)
Constant	5.1841	5.8826*	5.6177*	5.3897	–14.7799**	–14.5311**	–14.3666**	–14.2240**
	(2.6773)	(2.8124)	(2.7278)	(2.8687)	(5.1012)	(5.0729)	(4.9991)	(5.2182)
*n*	12,864	12,864	12,864	12,864	3,612	3,612	3,612	3,612

*Note:* Based on author’s calculations of data from Demographic and Health Surveys and Uppsala Conflict Data Program Georeferenced Event Dataset. All models adjust for birth order and the year of conception. Conflict frequency was measured as the number of battle events; conflict intensity was captured as the number of casualties in those events. Exposure to conflict is measured prior to conception. Municipality robust standard errors are in parentheses.

* *p* < .05, ** *p* < .01.

**Table 5.** Municipality Fixed Effects Linear Probability Models of Unwanted Births in Relation to Armed Conflict in Colombia, by the Respondent’s Type of Place of Residence (*N* = 16,216).

	Rural Area				Urban Area, Low Population Density				Urban Area, High Population Density			
	Model 1	Model 2	Model 3	Model 4	Model 5	Model 6	Model 7	Model 8	Model 9	Model 10	Model 11	Model 12
	*B* (*SE*)	*B* (*SE*)	*B* (*SE*)	*B* (*SE*)	*B* (*SE*)	*B* (*SE*)	*B* (*SE*)	*B* (*SE*)	*B* (*SE*)	*B* (*SE*)	*B* (*SE*)	*B* (*SE*)
Conflict frequency in the past year	–.0015(.0047)				–.0008(.0052)				.0048**(.0015)			
Conflict frequency in the past 5 years		.0022*				–.0012				.0008		
		(.0011)				(.0015)				(.0005)		
Conflict intensity in the past year			–.0019				.0001				.0019***	
			(.0012)				(.0004)				(.0004)	
Conflict intensity in the past 5 years				.0006*				–.0001				.0004*
				(.0003)				(.0002)				(.0002)
Constant	4.7659	1.4810	6.1870	1.8183	2.5540	3.4338	2.0702	2.8535	1.7686	3.7623	1.1690	3.1981
	(6.8657)	(7.1257)	(6.8008)	(7.0785)	(4.4032)	(4.5916)	(4.3695)	(5.1419)	(2.9060)	(2.8599)	(2.8446)	(2.8634)
*n*	4,723	4,723	4,723	4,723	4,121	4,121	4,121	4,121	7,372	7,372	7,372	7,372

*Note:* Based on author’s calculations of data from Demographic and Health Surveys and Uppsala Conflict Data Program Georeferenced Event Dataset. All models adjust for birth order, the year of conception, and the respondent’s age at conception. Conflict frequency was measured as the number of battle events; conflict intensity was captured as the number of casualties in those events. Exposure to conflict is measured prior to conception. Municipality robust standard errors are in parentheses.

* *p* < .05, **p < .01, ***p < .001.

**Table 6.** Municipality Fixed Effects Linear Probability Models of Unwanted Birth in Relation to Armed Conflict in Colombia, by the Respondent’s Type of Place of Residence (*N* = 16,476).

	Primary Education				Secondary Education				Tertiary Education			
	Model 1	Model 2	Model 3	Model 4	Model 5	Model 6	Model 7	Model 8	Model 9	Model 10	Model 11	Model 12
	*B* (*SE*)	*B* (*SE*)	*B* (*SE*)	*B* (*SE*)	*B* (*SE*)	*B* (*SE*)	*B* (*SE*)	*B* (*SE*)	*B* (*SE*)	*B* (*SE*)	*B* (*SE*)	*B* (*SE*)
Conflict frequency in the past year	.0042*				.0047*				–.0023			
	(.0019)				(.0023)				(.0029)			
Conflict frequency in the past 5 years		.0015				.0002				.0002		
		(.0008)				(.0007)				(.0010)		
Conflict intensity in the past year			.0007				.0013*				–.0005	
			(.0007)				(.0005)				(.0011)	
Conflict intensity in the past 5 years				.0004								.0002
				(.0002)				(.0002)				(.0003)
Constant	–5.9717	–5.5607	–5.0546	–5.6390	–4.2486	–1.8256	–4.0638	–2.6114	1.4044	–.1566	.9699	–1.1668
	(4.0473)	(4.0508)	(3.9993)	(4.0970)	(3.1408)	(3.2315)	(3.1587)	(3.3165)	(6.0208)	(6.2025)	(6.0074)	(6.3615)
*n*	7,541	7,541	7,541	7,541	6,727	6,727	6,727	6,727	2,208	2,208	2,208	2,208

*Note:* Based on author’s calculations of data from Demographic and Health Surveys and Uppsala Conflict Data Program Georeferenced Event Dataset. All models adjust for birth order, the year of conception, and the respondent’s age at conception. Conflict frequency was measured as the number of battle events; conflict intensity was captured as the number of casualties in those events. Exposure to conflict is measured prior to conception. Municipality robust standard errors are in parentheses.

* *p* < .05.

**Table 7.** Municipality Fixed Effects Linear Probability Models of Unwanted Births in Relation to Armed Conflict in Colombia, by Parity of the Child (*N* = 16,476).

	First Order				Second Order				Third or Higher Order			
	Model 1	Model 2	Model 3	Model 4	Model 5	Model 6	Model 7	Model 8	Model 9	Model 10	Model 11	Model 12
	*B* (*SE*)	*B* (*SE*)	*B* (*SE*)	*B* (*SE*)	*B* (*SE*)	*B* (*SE*)	*B* (*SE*)	*B* (*SE*)	*B* (*SE*)	*B* (*SE*)	*B* (*SE*)	*B* (*SE*)
Conflict frequency in the past year	.0032				.0049*				.0030			
	(.0019)				(.0019)				(.0025)			
Conflict frequency in the past 5 years		.0015*				.0013				–.0004		
		(.0006)				(.0007)				(.0007)		
Conflict intensity in the past year			.0008				.0013*				.0008	
			(.0006)				(.0005)				(.0006)	
Conflict intensity in the past 5 years				.0004*				.0004*				.0002
				(.0002)				(.0002)				(.0002)
Constant	–15.7428***	–16.3203***	–15.4245***	–16.5413***	–3.7291	–3.1570	–3.4244	–3.4066	5.2538	7.1682	5.3200	5.9547
	(3.4819)	(3.3413)	(3.3873)	(3.3875)	(3.5799)	(3.7275)	(3.8615)	(3.8374)	(4.0392)	(3.8644)	(4.0781)	(3.8516)
*n*	5,311	5,311	5,311	5,311	4,744	4,744	4,744	4,744	6,421	6,421	6,421	6,421

*Note:* Based on author’s calculations of data from Demographic and Health Surveys and Uppsala Conflict Data Program Georeferenced Event Dataset. All models adjust for the year of conception and the respondent’s age at conception. Conflict frequency was measured as the number of battle events; conflict intensity was captured as the number of casualties in those events. Exposure to conflict is measured prior to conception. Municipality robust standard errors are in parentheses.

* *p* < .05, ***p < .001.

Contrary to expectation, the only significant associations between conflict and unwanted births were found among respondents 20 years or older. Estimates for adolescents were positive but insignificant, indicating that conflict was unassociated with unwanted teenage fertility.

There were no significant associations in low-density urban areas, pointing toward rural areas and high-density urban areas as places where conflict limits reproductive autonomy and that suburban households are more resilient to conflict, perhaps reflecting higher socioeconomic status. This finding was robust to estimating 100 instead of 50 inhabitants per km^2^ as the cutoff for high population density (available upon request).

Significant associations were only found at secondary or lower levels of education—estimates among women with tertiary education were either negative or positive but consistently insignificant—again pointing toward higher socioeconomic status as a protective factor.

Significant relationships between exposure to conflict and unwanted birth were only observed at first and second—but not higher order—parities.

Finally, to address multiple marginalization (Scott and Siltanen 2017), I restricted the sample to respondents at the intersection of the social characteristics most likely to experience unwanted births when exposed to conflict. Intersecting these social characteristics (except age), I analyzed the relationship between conflict and unwanted first or second births among respondents who had secondary or lower levels of education. Respondents living in rural areas (n = 2,150) and high-density urban areas (n = 3,953) were examined separately. These respondents were exposed to a maximum of 419 casualties (residents in rural areas) or 272 casualties (residents in high-density urban areas) in the past five years.

Based on LPMs of these distinct intersections (see Appendix Table A6 in the online version of the article), the predicted probabilities of experiencing an unwanted birth among respondents who were unexposed to conflict in the past five years in rural and high-density areas ranged around 44 to 45 percentage points (CI ≈ 44–46).

By comparison, at the highest conflict levels in the past five years, the predicted probability of unwanted birth was 98 percentage points (CI = 67–130) in rural areas and 57 percentage points (CI = 49–65) in high-density urban areas. High-density urban residents were thus also vulnerable to conflict, although not as much as rural residents. Although these groups are selective, rurality stands out as a critical social characteristic that in conjunction with other attributes (lower levels of education and parity) shapes women’s reproductive experiences in the Colombian war.

### Robustness Checks

I estimated multiple respecifications of the main models (Table 2), available in Appendix Tables A6 to A13 in the online version of the article.

The direction, magnitude, and significance levels of estimates were largely consistent when (1) restricting the sample to the respondents’ latest births, (2) removing the covariates, and (3) adding the additional sociodemographic variables from the heterogeneous effects models as controls.

LPMs were preferred over logistic regression for cross-model comparisons and interpretability (Mood 2010). Logistic regressions with the same specifications confirmed a positive and significant association between conflict frequency in the past one year and unwanted birth, and the relationship with conflict intensity over the past five years was positive and insignificant. The consistency in one-year measures across both models reinforces the robustness of the main findings, although the differences in five-year indicators suggest less robust longer-term effects.

Fixed effects are advantageous over random effects because the latter assumes that unobserved variation captured in the area-specific error term is independent of all measured indicators (Angrist and Pischke 2009). Using random effects yielded estimates similar in direction and statistical significance, although slightly attenuated.

I used competing risk models to distinguish between whether births were wanted later (mistimed) or not at all (unwanted), in reference to being wanted. However, these models should be interpreted with caution due to social desirability bias (reporting a child as mistimed may be perceived as less stigmatizing than reporting a child as completely unwanted) and because multinomial regressions do not allow for fixed effects. These models revealed that conflict was associated with a higher relative risk of reporting children as mistimed but not completely unwanted.

I accounted for conflict spillovers from neighboring municipalities by adding spatial lags (for a technical description, see the Appendix in the online version of the article). Here, coefficients remained virtually identical, albeit significant at the 10% level, suggesting that although there is some spatial dependence, the relationship between conflict and unwanted births is not driven by spillover effects.

## Discussion

Colombia is at a watershed moment while finalizing the peace agreement implementation with the FARC-EP and burgeoning peace negotiations with the ELN. How the government responds to the impact of war on civilians will be a cornerstone of Colombia’s future development. Prior research has suggested a considerable negative impact of war on reproductive health in Colombia (Camacho 2008; Castro Torres and Urdinola 2018; Ramos Jaraba et al. 2020; Svallfors 2022a, 2022b, 2023, 2024c; Svallfors and Billingsley 2019; Svallfors et al. 2025) and the health care system (Crawford et al. 2023; Franco et al. 2006; Svallfors 2024c).

Adding to this literature, this study showed that exposure to conflict before conception correlated with a higher probability of experiencing unwanted birth among Colombian women. This was in line with expectations, guided by reproductive justice theory, because limited access to contraception and abortion alongside pervasive sexual violence are factors that severely restrain women’s reproductive autonomy (Hedström and Herder 2023; Svallfors 2024c). The findings suggest that fertility surges in conflict-affected rural areas of Colombia are likely explained, in part, by unwanted births (Castro Torres and Urdinola 2018).

When considering heterogeneous effects by sociodemographic characteristics, associations were driven by first and second births to socioeconomically and geographically marginalized women. This means that previous evidence of earlier first births among violence-affected Colombians may reflect voluntary fertility, perhaps due to fast-life history strategies in response to high mortality risks for young adult men (Nettle 2010; Svallfors 2024b). Although higher education and suburban residency appear to insulate women from the negative effects of conflict, women with less education in rural areas or urban slums may have less access to contraception or be more exposed to sexual violence (Svallfors 2024c). The results showed that intersecting dimensions of marginalization add up; multifold disadvantaged respondents were substantially more likely to experience an unwanted first or second birth when exposed to conflict compared to both their unexposed counterparts and the general population.

Although data limitations precluded analyses of other axes of marginalization and the deconstruction of socially molded categories, this study contributes to emerging discussions about intersectional dimensions of population health (Bauer 2014; Homan, Brown, and King 2021; Ross and Solinger 2017). As evidenced here, exposure to conflict is a contextual determinant that intersects with other dimensions of inequality in shaping health and bodily autonomy. This heterogeneity underscores the need for future research to consider intersectional—not only universal—patterns of how exposure to conflict shapes health.

Investigating various dimensions of conflict revealed little variation by conflict type, suggesting that violence itself—not who perpetrates or suffers from it—shapes reproductive autonomy during war. Conflict matters even when the battles do not involve civilians. Although the distinction between types of conflict is common in conflict research (Davies et al. 2023), this study is among the first to consider its importance for health.

Both conflict frequency and intensity in both more immediate and wider geographical surroundings were significantly linked to unwanted birth, corresponding to findings regarding detrimental mental health outcomes of more or less local violence in Mexico (Villarreal and Yu 2017). Conflict was only linked to unwanted birth when measured over intermediate or longer durations of time, suggesting that the erosion of health care resources adds up over time and/or that protracted conflict causes more sexual violence than short-term conflict.

Due to data limitations, only organized lethal violence was considered in this study. Conflict mortality by organized armed actors is a suitable measure for exposure to local conflict violence because the definition is comparable across settings and deadly violence may be more visible to citizens than, for example, landmine explosions (cf. Camacho 2008). UCDP-GED has been widely used in similar research (O’Brien 2020; Østby et al. 2018; Svallfors and Billingsley 2019; Torrisi 2022) and provides comprehensive coverage in Colombian departments and municipalities, although urban centers and areas with stronger media presence are more documented. Due to its reliance on media reports and exclusion of nonfatal events, UCDP-GED provides lower-bound estimates of the real extent of conflict violence.

It was impossible to investigate the role of contraception retrospectively, whether births resulted from coercive or consented sex, and how respondents perceived and responded to security threats. The sample was limited to live births, which is why no arguments can be made about unwanted pregnancies, only regarding unwanted births (Torrisi 2024; Weitzman et al. 2023). Assuming that many unwanted pregnancies result in miscarriage or abortion, estimates presented here are likely conservative.

Despite these drawbacks, the plausible explanations for these findings are relatively straightforward. Contraceptive prevalence is comparatively high in Colombia, a low-fertility context often discussed as a success story for family planning programs. Although the method mix varies considerably throughout the country, women exposed to high levels of conflict are one-third less likely to use any method of contraception compared to unexposed women, regardless of whether the method is short or long acting or user or provider administered (Svallfors and Billingsley 2019). This situation results from difficulties in providing health care in violent areas, restrictions put in place by armed groups, and reproductive governance in areas under these groups’ control (Svallfors 2024c).

All armed actors, including the military, have used sexual violence to punish political adversaries, sanction those who transgress essentialized gender norms, build group cohesion, and perform a militarized masculinity role (Svallfors 2024a, 2024c). Besides “conflict-related” sexual violence, women in more violent areas are more likely to be victimized by emotional, physical, and sexual partner violence (Svallfors 2023). Exposure to gender-based violence is a deeply disempowering experience that exposes women to the risk of unwanted pregnancy (Gomez 2011).

Although data limitations precluded evaluating these mechanisms empirically, the compounding effects of sexual violence, reproductive governance, limited access to contraception, and restricted bodily autonomy could explain why women—especially those in multifold marginalized social positions—experience unwanted births when exposed to conflict.

Retrospective reports about whether living children were wanted likely suffer from social desirability bias. Ex post rationalizations and dynamic or ambivalent fertility preferences challenge accurate estimates of unwanted pregnancy and birth (Mumford et al. 2016). However, there is no evidence in the literature of upward bias in retrospective reports of unwanted fertility. In this sample, a substantial share of respondents reported experiencing unwanted births; only 48% of live births were reported as wanted and well timed, 24% were wanted later, and 27% were not wanted at all. Moreover, the significant correlations between unwanted fertility and conflict exposure were consistently positive. Because both the exposure and outcome are likely underreported, the estimates should be considered floor effects.

The findings from this study extend beyond Colombia to illuminate broader patterns in how conflict affects reproductive autonomy globally. Although Colombia’s conflict has unique features, the core mechanisms identified here—health care disruption and increased sexual violence—are common across conflicts worldwide (Hedström and Herder 2023). This study suggests that conflict-induced fertility increases across diverse settings, such as Azerbaijan (Torrisi 2020), Burundi (Verwimp et al. 2020), and Cambodia (Islam et al. 2016), may partially reflect compromised reproductive autonomy rather than changed preferences. However, Colombia’s relatively strong health care system and high contraceptive prevalence make it a conservative case (Svallfors 2022a). The relationship between conflict and unwanted births may be even stronger in settings with weaker health infrastructure. Moreover, the findings may also extend beyond conflict to other forms of instability. Studies of criminal violence (Weitzman et al. 2023) and natural disasters (Nobles, Frankenberg, and Thomas 2015) suggest that similar mechanisms may operate across different crises. More research is needed to understand how various forms of instability interact with local contexts to shape reproductive outcomes.

This study adds to the literature on how sociodemographic factors—such as socioeconomic status, education, and access to health care—impact health (Wilkinson and Marmot 2003) by demonstrating how conflict operates as a structural determinant that interacts with and amplifies existing social gradients in health. Moreover, the study advances place-based perspectives on health (Bernard et al. 2007) and supports theories of how place and social position jointly determine health outcomes (Kawachi and Berkman 2003). By revealing how conflict compounds existing inequities, the study contributes to intersectional approaches examining how multiple forms of disadvantage combine to affect reproductive autonomy (Bauer 2014). This aligns with reproductive justice theory highlighting how sociopolitical contexts shape lived experiences of reproductive well-being differently across social groups (Ross and Solinger 2017).

This study also advances knowledge of the predictors of unwanted pregnancy and birth (D’Angelo et al. 2004). Conflict emerges as a contextual determinant—related to politics, economics, and social norms—stratifying women’s ability to exercise reproductive choice (Ross and Solinger 2017). Past research shows that although unintended pregnancy is not associated with adverse health for those who perceive it as a positive surprise, it may cause substantial long-term health penalties for those who react more negatively (Yeatman and Smith-Greenaway 2021).

The results underscore the need for governmental, humanitarian, and transitional justice programs to support war-affected women’s reproductive autonomy. Such initiatives may include strengthening Colombia’s comprehensive but war-affected family planning programs (Svallfors 2024c; Svallfors and Billingsley 2019). After recently decriminalizing abortion, the availability, accessibility, affordability, and quality of abortion and postabortion complication services must be secured in Colombia (Daigle et al. 2022). Additional support is needed in rural areas and socioeconomically disadvantaged populations. Women who experience an unwanted pregnancy or birth should be provided with psychosocial, antenatal, postnatal, childrearing, and socioeconomic support as needed. Ultimately, supporting sexual and reproductive health aligns with the goals of positive peace: reducing social inequality and arms proliferation that could cause violent reescalation. Research and advocacy are critical to elucidate the deleterious effects of conflict on health and demonstrate why peacebuilding matters for long-term population prosperity. This study contributes to that effort.

## Supplemental Material

sj-pdf-1-hsb-10.1177_00221465251353533 – Supplemental material for Armed Conflict and Unwanted Births in Colombia Supplemental material, sj-pdf-1-hsb-10.1177_00221465251353533 for Armed Conflict and Unwanted Births in Colombia by Signe Svallfors in Journal of Health and Social Behavior
