# Supplementary material for: Armed Conflict and Unwanted Births in Colombia
Source: J Health Soc Behav. 2025 Jul 24;67(2):290–309. doi: 10.1177/00221465251353533 (PMC13219790; doi:10.1177/00221465251353533)
Supplement: sj-pdf-1-hsb-10.1177_00221465251353533 – Supplemental material for Armed Conflict and Unwanted Births in Colombia [file sj-pdf-1-hsb-10.1177_00221465251353533.pdf]

Journal of Health and Social Behavior

OFFICIAL JOURNAL OF THE AMERICAN SOCIOLOGICAL ASSOCIATION

ONLINE SUPPLEMENT

to article in

Journal of Health and Social Behavior

Armed Conflict and Unwanted Births in Colombia

Signe Svallfors
Stockholm University
Stanford University

Table of contents:

1. Models using alternative specifications of conflict exposure: Tables A1–A4
2. Additional descriptive statistics of the sample population: Table A5
3. Intersectional models of first or second births among respondents with secondary or lower levels of education, comparing residents of rural and high-density urban areas: Table A6
4. Models including only the latest birth to each respondent to account for potential serial correlation between births: Table A7
5. Competing risk models differentiating between mistimed and completely unwanted births: Table A8
6. Logistic regression models: Table A9
7. Crude models without covariates: Table A10
8. Models additionally controlling for education, type of residence, and parity: Table A11
9. Models substituting fixed with random effects: Table A12
10. Technical description of spatial weights
11. Models adding spatial weights for conflict exposure: Table A13

Table A1. Municipality-fixed effects linear probability models of unwanted birth in relation to armed conflict in Colombia, using alternative specifications of conflict exposure

	**Model 1**	**Model 2**	**Model 3**	**Model 4**	**Model 5**	**Model 6**	**Model 7**	**Model 8**
	B/SE	B/SE	B/SE	B/SE	B/SE	B/SE	B/SE	B/SE
**Department-level indicators**								
Conflict frequency in the past month	0.00099							
	(0.0007)							
Conflict frequency in the past 3 months		0.00054*						
		(0.0002)						
Conflict frequency in the past year			0.00025***					
			(0.0000)					
Conflict frequency in the past 5 years				0.00009*				
				(0.0000)				
Conflict intensity in the past month					0.00021			
					(0.0002)			
Conflict intensity in the past 3 months						0.00028***		
						(0.0000)		
Conflict intensity in the past year							0.00009***	
							(0.0000)	
Conflict intensity in the past 5 years								0.00003**
								(0.0000)
Constant	3.08752	2.75576	2.07461	2.11303	3.26116	2.14939	1.69543	1.55922
	(2.3581)	(2.3303)	(2.2930)	(2.3970)	(2.5004)	(2.3596)	(2.2449)	(2.3359)
N	16476	16476	16476	16476	16476	16476	16476	16476
R2	0.01	0.01	0.01	0.01	0.01	0.01	0.01	0.01
AIC	23683.10	23682.29	23679.15	23675.91	23683.38	23679.30	23677.43	23674.59

* p<0.05, ** p<0.01, *** p<0.001. All models adjust for birth order, the year of conception, and the respondent's age at conception. Conflict frequency was measured as the number of battle events; conflict intensity was captured as the number of casualties in those events. Exposure to conflict is measured prior to conception. Municipality-robust standard errors in brackets.

Table A1. Continued

	Model 9	Model 10	Model 11	Model 12	Model 13	Model 14	Model 15	Model 16
	B/SE	B/SE	B/SE	B/SE	B/SE	B/SE	B/SE	B/SE
**Municipality-level indicators**								
Conflict frequency in the past month	0.00178							
	(0.0106)							
Conflict frequency in the past 3 months		0.00463						
		(0.0050)						
Conflict frequency in the past year			0.00358*					
			(0.0014)					
Conflict frequency in the past 5 years				0.00084				
				(0.0005)				
Conflict intensity in the past month					-0.00082			
					(0.0022)			
Conflict intensity in the past 3 months						0.00068		
						(0.0012)		
Conflict intensity in the past year							0.00078	
							(0.0005)	
Conflict intensity in the past 5 years								0.00028*
								(0.0001)
Constant	2.71095	2.14904	0.89898	1.62102	2.92296	2.49640	1.40309	1.24553
	(2.4979)	(2.4648)	(2.4150)	(2.4964)	(2.4723)	(2.3854)	(2.4143)	(2.5428)
N	16476	16476	16476	16476	16476	16476	16476	16476
R2	0.01	0.01	0.01	0.01	0.01	0.01	0.01	0.01
AIC	23100.02	23099.03	23094.42	23096.88	23099.89	23099.70	23096.39	23096.18

* $p<0.05$, ** $p<0.01$, *** $p<0.001$. All models adjust for birth order, the year of conception, and the respondent's age at conception. Conflict frequency was measured as the number of battle events; conflict intensity was captured as the number of casualties in those events. Exposure to conflict is measured prior to conception. Municipality-robust standard errors in brackets.

Table A2. Fixed effects linear probability models of unwanted birth in relation to armed conflict in Colombia, using alternative specifications of state-based conflict

	**Model 1**	**Model 2**	**Model 3**	**Model 4**	**Model 5**	**Model 6**	**Model 7**	**Model 8**
	B/SE	B/SE	B/SE	B/SE	B/SE	B/SE	B/SE	B/SE
**Department-level indicators**								
Conflict frequency in the past month	0.00285*							
	(0.0012)							
Conflict frequency in the past 3 months		0.00163**						
		(0.0005)						
Conflict frequency in the past year			0.00071***					
			(0.0001)					
Conflict frequency in the past 5 years				0.00017*				
				(0.0001)				
Conflict intensity in the past month					-0.00010			
					(0.0003)			
Conflict intensity in the past 3 months						0.00052**		
						(0.0002)		
Conflict intensity in the past year							0.00020***	
							(0.0000)	
Conflict intensity in the past 5 years								0.00006**
								(0.0000)
Constant	2.92897	2.43506	1.68787	2.63962	3.70181	2.33343	1.67655	2.02231
	(2.2957)	(2.2327)	(2.2498)	(2.4640)	(2.4644)	(2.2197)	(2.1371)	(2.3280)
N	16476	16476	16476	16476	16476	16476	16476	16476
R2 overall	0.01	0.01	0.01	0.01	0.01	0.01	0.01	0.01
AIC	23682.48	23680.95	23677.02	23678.69	23683.73	23679.39	23677.47	23678.24

* p<0.05, ** p<0.01, *** p<0.001. All models adjust for birth order, the year of conception, and the respondent's age at conception. Conflict frequency was measured as the number of battle events; conflict intensity was captured as the number of casualties in those events. Exposure to conflict is measured prior to conception. Municipality-robust standard errors in brackets. Municipality-robust standard errors in brackets.

Table A2. Continued

	Model 9	Model 10	Model 11	Model 12	Model 13	Model 14	Model 15	Model 16
	B/SE	B/SE	B/SE	B/SE	B/SE	B/SE	B/SE	B/SE
**Municipality-level indicators**								
Conflict frequency in the past month	0.02443							
	(0.0160)							
Conflict frequency in the past 3 months		0.01327						
		(0.0074)						
Conflict frequency in the past year			0.00785***					
			(0.0023)					
Conflict frequency in the past 5 years				0.00066				
				(0.0011)				
Conflict intensity in the past month					-0.00192			
					(0.0029)			
Conflict intensity in the past 3 months						0.00071		
						(0.0015)		
Conflict intensity in the past year							0.00116*	
							(0.0005)	
Conflict intensity in the past 5 years								0.00031
								(0.0002)
Constant	2.30714	1.98005	0.96778	2.37112	2.95452	2.62414	1.59837	1.70915
	(2.4606)	(2.4116)	(2.4088)	(2.4761)	(2.4950)	(2.4297)	(2.4026)	(2.5598)
N	16476	16476	16476	16476	16476	16476	16476	16476
R2 overall	0.01	0.01	0.01	0.01	0.01	0.01	0.01	0.01
AIC	23097.33	23096.95	23091.85	23099.58	23099.53	23099.84	23095.84	23098.09

* p<0.05, ** p<0.01, *** p<0.001. All models adjust for birth order, the year of conception, and the respondent's age at conception. Conflict frequency was measured as the number of battle events; conflict intensity was captured as the number of casualties in those events. Exposure to conflict is measured prior to conception. Municipality-robust standard errors in brackets. Municipality-robust standard errors in brackets.

Table A3. Fixed effects linear probability models of unwanted birth in relation to armed conflict in Colombia, using alternative specifications of non-state conflict

	**Model 1**	**Model 2**	**Model 3**	**Model 4**	**Model 5**	**Model 6**	**Model 7**	**Model 8**
	B/SE	B/SE	B/SE	B/SE	B/SE	B/SE	B/SE	B/SE
**Department-level indicators**								
Conflict frequency in the past month	0.00205							
	(0.0120)							
Conflict frequency in the past 3 months		0.00557						
		(0.0050)						
Conflict frequency in the past year			0.00341***					
			(0.0008)					
Conflict frequency in the past 5 years				0.00167**				
				(0.0006)				
Conflict intensity in the past month					0.00089			
					(0.0008)			
Conflict intensity in the past 3 months						0.00075**		
						(0.0002)		
Conflict intensity in the past year							0.00037***	
							(0.0001)	
Conflict intensity in the past 5 years								0.00014***
								(0.0000)
Constant	3.57595	3.21865	2.63495	2.16952	3.43690	3.14531	2.59793	2.36541
	(2.5138)	(2.3801)	(2.2715)	(2.3536)	(2.4546)	(2.4085)	(2.2972)	(2.2738)
N	16476	16476	16476	16476	16476	16476	16476	16476
R2	0.01	0.01	0.01	0.01	0.01	0.01	0.01	0.01
AIC	23683.73	23682.92	23680.26	23676.42	23682.93	23681.35	23678.47	23677.77

* p<0.05, ** p<0.01, *** p<0.001. All models adjust for birth order, the year of conception, and the respondent's age at conception. Conflict frequency was measured as the number of battle events; conflict intensity was captured as the number of casualties in those events. Exposure to conflict is measured prior to conception. Municipality-robust standard errors in brackets.

Table A3. Continued

	**Model 9**	**Model 10**	**Model 11**	**Model 12**	**Model 13**	**Model 14**	**Model 15**	**Model 16**
	B/SE	B/SE	B/SE	B/SE	B/SE	B/SE	B/SE	B/SE
**Municipality-level indicators**								
Conflict frequency in the past month	-0.08535*							
	(0.0367)							
Conflict frequency in the past 3 months		-0.05839**						
		(0.0180)						
Conflict frequency in the past year			0.00365					
			(0.0146)					
Conflict frequency in the past 5 years				0.00933*				
				(0.0040)				
Conflict intensity in the past month					-0.01027			
					(0.0170)			
Conflict intensity in the past 3 months						-0.00140		
						(0.0034)		
Conflict intensity in the past year							-0.00079	
							(0.0016)	
Conflict intensity in the past 5 years								0.00240*
								(0.0011)
Constant	2.98580	3.17831	2.71145	2.20385	2.86987	2.83517	2.88525	1.85290
	(2.5437)	(2.5939)	(2.4936)	(2.4957)	(2.5284)	(2.5303)	(2.5358)	(2.5089)
N	16476	16476	16476	16476	16476	16476	16476	16476
R2	0.01	0.01	0.01	0.01	0.01	0.01	0.01	0.01
AIC	23097.46	23096.51	23099.98	23097.29	23099.35	23099.94	23099.89	23094.09

* p<0.05, ** p<0.01, *** p<0.001. All models adjust for birth order, the year of conception, and the respondent's age at conception. Conflict frequency was measured as the number of battle events; conflict intensity was captured as the number of casualties in those events. Exposure to conflict is measured prior to conception. Municipality-robust standard errors in brackets.

Table A4. Fixed effects linear probability models of unwanted birth in relation to armed conflict in Colombia, using alternative specifications of one-sided conflict

	**Model 1**	**Model 2**	**Model 3**	**Model 4**	**Model 5**	**Model 6**	**Model 7**	**Model 8**
	B/SE	B/SE	B/SE	B/SE	B/SE	B/SE	B/SE	B/SE
**Department-level indicators**								
Conflict frequency in the past month	0.00068							
	(0.0015)							
Conflict frequency in the past 3 months		0.00039						
		(0.0004)						
Conflict frequency in the past year			0.00026***					
			(0.0001)					
Conflict frequency in the past 5 years				0.00016**				
				(0.0001)				
Conflict intensity in the past month					0.00053			
					(0.0005)			
Conflict intensity in the past 3 months						0.00029*		
						(0.0001)		
Conflict intensity in the past year							0.00012***	
							(0.0000)	
Conflict intensity in the past 5 years								0.00007**
								(0.0000)
Constant	3.43682	3.30309	2.82130	2.16205	3.24278	2.99531	2.61129	1.60558
	(2.3796)	(2.3869)	(2.3245)	(2.3361)	(2.4118)	(2.4206)	(2.3127)	(2.2996)
N	16476	16476	16476	16476	16476	16476	16476	16476
R2	0.01	0.01	0.01	0.01	0.01	0.01	0.01	0.01
AIC	23683.62	23683.42	23681.76	23676.10	23683.15	23682.50	23681.02	23673.08

* p<0.05, ** p<0.01, *** p<0.001. All models adjust for birth order, the year of conception, and the respondent’s age at conception. Conflict frequency was measured as the number of battle events; conflict intensity was captured as the number of casualties in those events. Exposure to conflict is measured prior to conception. Municipality-robust standard errors in brackets.

Table A4. Continued

	Model 9	Model 10	Model 11	Model 12	Model 13	Model 14	Model 15	Model 16
	B/SE	B/SE	B/SE	B/SE	B/SE	B/SE	B/SE	B/SE
**Municipality-level indicators**								
Conflict frequency in the past month	-0.01210							
	(0.0152)							
Conflict frequency in the past 3 months		0.00224						
		(0.0064)						
Conflict frequency in the past year			0.00292					
			(0.0026)					
Conflict frequency in the past 5 years				0.00172*				
				(0.0009)				
Conflict intensity in the past month					0.00166			
					(0.0025)			
Conflict intensity in the past 3 months						0.00120		
						(0.0020)		
Conflict intensity in the past year							0.00090	
							(0.0010)	
Conflict intensity in the past 5 years								0.00042
								(0.0003)
Constant	3.11409	2.63607	2.00041	1.61338	2.68908	2.58870	2.21505	2.10964
	(2.5253)	(2.4915)	(2.4673)	(2.5665)	(2.5012)	(2.4332)	(2.4222)	(2.6019)
N	16476	16476	16476	16476	16476	16476	16476	16476
R2	0.01	0.01	0.01	0.01	0.01	0.01	0.01	0.01
AIC	23099.31	23099.95	23098.58	23095.29	23099.81	23099.68	23098.91	23097.94

* p<0.05, ** p<0.01, *** p<0.001. All models adjust for birth order, the year of conception, and the respondent's age at conception. Conflict frequency was measured as the number of battle events; conflict intensity was captured as the number of casualties in those events. Exposure to conflict is measured prior to conception. Municipality-robust standard errors in brackets.

Table A5. Additional descriptive statistics of the sample population (n = 16,476)

	**Frequency/mean**	**Proportion/SD**
Exposure to all types of violence		
Conflict frequency in the past month	0.10	0.43
Conflict frequency in the past 3 months	0.28	0.94
Conflict intensity in the past month	0.29	1.93
Conflict intensity in the past 3 months	0.88	3.65
Exposure to state-based violence		
Conflict frequency in the past month	0.05	0.27
Conflict frequency in the past 3 months	0.14	0.55
Conflict frequency in the past year	0.60	1.68
Conflict frequency in the past 5 years	3.43	6.38
Conflict intensity in the past month	0.17	1.46
Conflict intensity in the past 3 months	0.50	2.60
Conflict intensity in the past year	2.16	7.59
Conflict intensity in the past 5 years	12.37	25.15
Exposure to non-state violence		
Conflict frequency in the past month	0.004	0.07
Conflict frequency in the past 3 months	0.01	0.13
Conflict frequency in the past year	0.04	0.31
Conflict frequency in the past 5 years	0.23	0.90
Conflict intensity in the past month	0.01	0.31
Conflict intensity in the past 3 months	0.05	0.97
Conflict intensity in the past year	0.23	2.09
Conflict intensity in the past 5 years	1.16	5.17
Exposure to one-sided violence		
Conflict frequency in the past month	0.04	0.28
Conflict frequency in the past 3 months	0.13	0.60
Conflict frequency in the past year	0.52	1.83
Conflict frequency in the past 5 years	2.84	6.54
Conflict intensity in the past month	0.12	1.15
Conflict intensity in the past 3 months	0.33	2.03
Conflict intensity in the past year	1.30	5.11
Conflict intensity in the past 5 years	7.25	18.06

Table A6. Municipality-fixed effects linear probability models of unwanted birth in relation to armed conflict in Colombia, selecting first or second births to respondents with secondary education or lower

**Residents of rural areas**	**Model 1**	**Model 2**	**Model 3**	**Model 4**
	B/SE	B/SE	B/SE	B/SE
Conflict frequency in the past year	0.0016			
	(0.0083)			
Conflict frequency in the past 5 years		**0.0046****		
		(0.0015)		
Conflict intensity in the past year			-0.0001	
			(0.0016)	
Conflict intensity in the past 5 years				**0.0013****
				(0.0004)
Constant	-6.4073	-12.6059	-5.7456	-11.8748
	(8.0659)	(8.1588)	(7.8627)	(8.1218)
N	2150	2150	2150	2150
R2	0.04	0.04	0.04	0.04
Rho	0.30	0.30	0.30	0.30
AIC	2481.23	2473.65	2481.30	2474.16
**Residents of high-density urban areas**	**Model 5**	**Model 6**	**Model 7**	**Model 8**
	B/SE	B/SE	B/SE	B/SE
Conflict frequency in the past year	**0.0055****			
	(0.0017)			
Conflict frequency in the past 5 years		0.0011		
		(0.0007)		
Conflict intensity in the past year			**0.0017*****	
			(0.0005)	
Conflict intensity in the past 5 years				**0.0005***
				(0.0002)
Constant	-11.0669***	-8.9157**	-10.8856**	-9.6666**
	(3.2704)	(3.2771)	(3.3486)	(3.2534)
N	3953	3953	3953	3953
R2	0.06	0.06	0.06	0.06
Rho	0.27	0.27	0.27	0.27
AIC	5237.57	5240.97	5237.98	5239.78

* p<0.05, ** p<0.01, *** p<0.001. All models adjust for birth order, the year of conception, and the respondent's age at conception. Conflict frequency was measured as the number of battle events; conflict intensity was captured as the number of casualties in those events. Exposure to conflict is measured prior to conception. Municipality-robust standard errors in brackets.

Table A7. Municipality-fixed effects linear probability models of unwanted birth in relation to armed conflict in Colombia, latest births only

	**Model 1**	**Model 2**	**Model 3**	**Model 4**
	B/SE	B/SE	B/SE	B/SE
Conflict frequency in the past year	**0.0037***			
	(0.0015)			
Conflict frequency in the past 5 years		0.0004		
		(0.0005)		
Conflict intensity in the past year			**0.0012****	
			(0.0004)	
Conflict intensity in the past 5 years				0.0002
				(0.0001)
Constant	2.2183	3.6694	2.1593	3.1679
	(2.6730)	(2.7573)	(2.6552)	(2.7997)
N	13873	13873	13873	13873

* p<0.05, ** p<0.01, *** p<0.001. All models adjust for the year of conception and the respondent's age at conception. Conflict frequency was measured as the number of battle events; conflict intensity was captured as the number of casualties in those events. Exposure to conflict is measured prior to conception. Municipality-robust standard errors in brackets.

Table A8. Competing risk models of unwanted and mistimed birth in relation to armed conflict in Colombia

	Model 1	Model 2	Model 3	Model 4
	RRR/SE	RRR/SE	RRR/SE	RRR/SE
**Mistimed births**				
Conflict frequency in the past year	1.012			
	(0.007)			
Conflict frequency in the past 5 years		**1.004***		
		(0.002)		
Conflict intensity in the past year			1.002	
			(0.003)	
Conflict intensity in the past 5 years				**1.001****
				(0.001)
Constant	0.000	0.000	0.000	0.000
	(0.000)	(0.000)	(0.000)	(0.000)
**Completely unwanted births**				
Conflict frequency in the past year	1.008			
	(0.006)			
Conflict frequency in the past 5 years		1.000		
		(0.002)		
Conflict intensity in the past year			1.002	
			(0.001)	
Conflict intensity in the past 5 years				1.000
				(0.001)
Constant	4.052e+10*	2.564e+12**	2.983e+10*	1.818e+12*
	(4.431e+11)	(2.794e+13)	(3.308e+11)	(2.051e+13)
N	16476	16476	16476	16476

* p<0.05, ** p<0.01, *** p<0.001. All models adjust for birth order, the year of conception, and the respondent's age at conception. Conflict frequency was measured as the number of battle events; conflict intensity was captured as the number of casualties in those events. Exposure to conflict is measured prior to conception. Base category: wanted births. Coefficients are exponentiated. Municipality-robust standard errors in brackets.

Table A9. Municipality-fixed effects logistic regression models of unwanted births in relation to armed conflict in Colombia

	**Model 1** OR/SE	**Model 2** OR/SE	**Model 3** OR/SE	**Model 4** OR/SE
Conflict frequency in the past year	**1.0147*** (0.0064)			
Conflict frequency in the past 5 years		1.0034 (0.0019)		
Conflict intensity in the past year			1.0032 (0.0017)	
Conflict intensity in the past 5 years				1.0011 (0.0006)
N	16361	16361	16361	16361

* p<0.05, ** p<0.01, *** p<0.001. All models adjust for birth order, the year of conception, and the respondent's age at conception. Conflict frequency was measured as the number of battle events; conflict intensity was captured as the number of casualties in those events. Exposure to conflict is measured prior to conception. Coefficients are exponentiated. Municipality-robust standard errors in brackets.

Table A10. Municipality-fixed effects linear probability models of unwanted birth in relation to armed conflict in Colombia, crude models

	**Model 1**	**Model 2**	**Model 3**	**Model 4**
	B/SE	B/SE	B/SE	B/SE
Conflict frequency in the past year	**0.0036***			
	(0.0014)			
Conflict frequency in the past 5 years		0.0007		
		(0.0005)		
Conflict intensity in the past year			0.0008	
			(0.0005)	
Conflict intensity in the past 5 years				**0.0002***
				(0.0001)
Constant	0.5153***	0.5147***	0.5166***	0.5146***
	(0.0017)	(0.0029)	(0.0019)	(0.0024)
N	16476	16476	16476	16476
R2	0.00	0.00	0.00	0.00
Rho	0.15	0.15	0.15	0.15
AIC	23208.89	23212.64	23211.19	23212.12

* p<0.05, ** p<0.01, *** p<0.001. Conflict frequency was measured as the number of battle events; conflict intensity was captured as the number of casualties in those events. Exposure to conflict is measured prior to conception. Municipality-robust standard errors in brackets.

Table A11. Municipality-fixed effects linear probability models of unwanted birth in relation to armed conflict in Colombia, including additional sociodemographic covariates

	**Model 1**	**Model 2**	**Model 3**	**Model 4**
	B/SE	B/SE	B/SE	B/SE
Conflict frequency in the past year	**0.0032***			
	(0.0013)			
Conflict frequency in the past 5 years		0.0008		
		(0.0004)		
Conflict intensity in the past year			0.0008*	
			(0.0004)	
Conflict intensity in the past 5 years				**0.0003***
				(0.0001)
Constant	-6.3466**	-5.6925*	-6.0666**	-6.1939**
	(2.2930)	(2.3017)	(2.2936)	(2.3629)
N	16476	16476	16476	16476
R2	0.09	0.09	0.09	0.09
Rho	0.15	0.15	0.15	0.15
AIC	21622.67	21624.86	21623.46	21623.39

* p<0.05, ** p<0.01, *** p<0.001. All models adjust for birth order, the year of conception, and the respondent's age at conception, educational level, type of place of residence, and parity. Conflict frequency was measured as the number of battle events; conflict intensity was captured as the number of casualties in those events. Exposure to conflict is measured prior to conception.

Table A12. Municipality-random effects linear probability models of unwanted birth in relation to armed conflict in Colombia

	**Model 1**	**Model 2**	**Model 3**	**Model 4**
	B/SE	B/SE	B/SE	B/SE
Conflict frequency in the past year	**0.0029***			
	(0.0014)			
Conflict frequency in the past 5 years		0.0006		
		(0.0004)		
Conflict intensity in the past year			0.0007	
			(0.0005)	
Conflict intensity in the past 5 years				**0.0002***
				(0.0001)
Constant	1.4281	2.0558	1.7549	1.6709
	(2.0585)	(2.1129)	(2.0624)	(2.1349)
N	16476	16476	16476	16476
Rho	0.03	0.03	0.03	0.03

* $p<0.05$, ** $p<0.01$, *** $p<0.001$. All models adjust for birth order, the year of conception, and the respondent's age at conception. Conflict frequency was measured as the number of battle events; conflict intensity was captured as the number of casualties in those events. Exposure to conflict is measured prior to conception. Municipality-robust standard errors in brackets.

Technical description of spatial weights

I accounted for potential spatial spillovers in conflict exposure by calculating spatial lags of conflict events and casualties. I constructed a spatial weights matrix using queen contiguity, which defines neighboring municipalities as those sharing any part of their boundary (even a single point). For each municipality-month observation, I calculated the weighted average of conflict events and deaths in neighboring municipalities, with weights row-standardized to ensure equal weighting of all neighbors.

The spatial analysis revealed that the Colombian municipal landscape consists of three distinct sub-graphs: one main network of 1,120 interconnected mainland municipalities and two isolated municipalities. These isolated units are San Andrés and Providencia, two Caribbean islands that have no contiguous neighbors and historically experienced no conflict events. For these isolated municipalities, spatial lags were set to zero, reflecting their geographical separation from the mainland conflict dynamics.

Analysis of spatial correlation reveals significant spillover patterns in conflict events across municipal boundaries. The correlation between a municipality's conflict events and the average events in neighboring municipalities is 0.17 (Pearson) to 0.20 (Spearman), suggesting meaningful spatial clustering of violent incidents. However, the spatial correlation in conflict deaths is notably weaker (Pearson = 0.02, Spearman = 0.03), indicating that while conflict activities tend to cluster geographically, their lethality does not follow the same spatial pattern. Visual inspection of scatter plots of these relationships confirms this pattern: while conflict events show clear positive spatial association, conflict deaths are largely concentrated near zero with sporadic extreme events that show no clear spatial pattern.

Table A13. Municipality-fixed effects linear probability models of unwanted birth in relation to armed conflict in Colombia, adding spatial weights for conflict exposure

	**Model 1** B/SE	**Model 2** B/SE	**Model 3** B/SE	**Model 4** B/SE
Conflict frequency in the past year	**0.0035+**			
	(0.0018)			
Conflict frequency in the past 5 years		0.0007		
		(0.0005)		
Conflict intensity in the past year			0.0006	
			(0.0006)	
Conflict intensity in the past 5 years				**0.0003+**
				(0.0001)
Constant	0.8948	1.6150	1.1993	1.2221
	(2.4209)	(2.4997)	(2.4348)	(2.5588)
N	16476	16476	16476	16476
R2	0.01	0.01	0.01	0.01
Rho	0.16	0.16	0.16	0.16
AIC	23096.42	23098.74	23097.94	23098.16

+ p<0.10, * p<0.05, ** p<0.01, *** p<0.001. All models adjust for birth order, the year of conception, and the respondent's age at conception. Conflict frequency was measured as the number of battle events; conflict intensity was captured as the number of casualties in those events. Exposure to conflict is measured prior to conception. Municipality-robust standard errors in brackets.
